# Outcomes of Hospitalized Patients With COVID-19 With Acute Kidney Injury and Acute Cardiac Injury

**DOI:** 10.3389/fcvm.2021.798897

**Published:** 2022-02-15

**Authors:** Justin Y. Lu, Alexandra Buczek, Roman Fleysher, Wouter S. Hoogenboom, Wei Hou, Carlos J. Rodriguez, Molly C. Fisher, Tim Q. Duong

**Affiliations:** ^1^Department of Radiology, Montefiore Medical Center, Albert Einstein College of Medicine, Bronx, NY, United States; ^2^Department of Family, Population and Preventive Medicine, Stony Brook Medicine, New York, NY, United States; ^3^Cardiology Division, Department of Medicine, Montefiore Medical Center, Albert Einstein College of Medicine, Bronx, NY, United States; ^4^Nephrology Division, Department of Medicine, Montefiore Medical Center, Albert Einstein College of Medicine, Bronx, NY, United States

**Keywords:** SARS-CoV-2, cardiovascular sequelae, cardiac injury, predictive model, AKI

## Abstract

**Purpose:**

This study investigated the incidence, disease course, risk factors, and mortality in COVID-19 patients who developed both acute kidney injury (AKI) and acute cardiac injury (ACI), and compared to those with AKI only, ACI only, and no injury (NI).

**Methods:**

This retrospective study consisted of hospitalized COVID-19 patients at Montefiore Health System in Bronx, New York between March 11, 2020 and January 29, 2021. Demographics, comorbidities, vitals, and laboratory tests were collected during hospitalization. Predictive models were used to predict AKI, ACI, and AKI-ACI onset. Longitudinal laboratory tests were analyzed with time-lock to discharge alive or death.

**Results:**

Of the 5,896 hospitalized COVID-19 patients, 44, 19, 9, and 28% had NI, AKI, ACI, and AKI-ACI, respectively. Most ACI presented very early (within a day or two) during hospitalization in contrast to AKI (*p* < 0.05). Patients with combined AKI-ACI were significantly older, more often men and had more comorbidities, and higher levels of cardiac, kidney, liver, inflammatory, and immunological markers compared to those of the AKI, ACI, and NI groups. The adjusted hospital-mortality odds ratios were 17.1 [95% CI = 13.6–21.7, *p* < 0.001], 7.2 [95% CI = 5.4–9.6, *p* < 0.001], and 4.7 [95% CI = 3.7–6.1, *p* < 0.001] for AKI-ACI, ACI, and AKI, respectively, relative to NI. A predictive model of AKI-ACI onset using top predictors yielded 97% accuracy. Longitudinal laboratory data predicted mortality of AKI-ACI patients up to 5 days prior to outcome, with an area-under-the-curve, ranging from 0.68 to 0.89.

**Conclusions:**

COVID-19 patients with AKI-ACI had markedly worse outcomes compared to those only AKI, ACI and NI. Common laboratory variables accurately predicted AKI-ACI. The ability to identify patients at risk for AKI-ACI could lead to earlier intervention and improvement in clinical outcomes.

## Introduction

Acute kidney injury (AKI) and acute cardiac injury (ACI) are well-recognized complications of coronavirus disease 2019 (COVID-19) caused by the severe acute respiratory syndrome coronavirus 2 (SARS-CoV-2) (1–3). AKI and ACI separately have been associated with increased risk of critical illness and mortality in COVID-19 patients (1–3). The mechanisms underlying the high incidence of AKI and ACI and their association with poor outcomes in COVID-19 are not well-understood and are likely multifactorial. SARS-CoV-2 uses the angiotensin-converting enzyme 2 (ACE2) as docking and entry receptor on host cells, and the transmembrane serine protease 2 (TMPRSS2) is also involved in its cellular entry (4, 5). Though unproven, it has been hypothesized that SARS-CoV2 may directly induce AKI and ACI as the kidney and heart have a high density of ACE2 receptors. Indirect effects of COVID-19 that contribute to AKI and ACI include hypoxia, hypotension, inflammation, thromboembolism, cytokine storm, and sepsis (1–3, 6, 7). Endothelial dysfunction has been reported in patients with severe COVID-19 (8) and also likely plays a role in AKI and ACI.

In addition to age, pre-existing hypertension, diabetes, and obesity are major risk factors for severe COVID-19 and increased mortality (9–11). Black and Hispanic patients have been disproportionately affected by COVID-19 and have increased mortality. This may be due to a higher prevalence of cardiovascular risk factors in this population or socioeconomic factors such as crowding, food insecurity and poverty (12–15).

Observational studies have characterized the risk factors and outcomes of AKI (16–21) and ACI (22–25) separately among hospitalized patients with COVID-19. However, there have been no systematic studies comparing outcomes of COVID-19 patients with AKI to COVID-19 patients with ACI or evaluating the incidence, risk factors and clinical outcomes of COVID-19 patients who develop both AKI and ACI during hospitalization. Understanding the clinical characteristics and risk factors that make COVID-19 patients susceptible to in-hospital AKI and ACI could lead to better patient management and clinical outcomes.

The purpose of this study was to investigate the demographics and the clinical variables of COVID-19 patients with combined injury (AKI-ACI), and to compare them with those with AKI only, ACI only, and no injury (NI). Our study population came from Montefiore Health System in the Bronx, New York, which serves a large, low-income, and diverse population and which was hit hard by the COVID-19 pandemic. Mathematical models were developed to predict AKI-ACI onset. In addition, we analyzed the temporal progression of different clinical variables with time-lock to outcome (discharged alive or in-hospital death) and use them to predict likelihood of mortality. To our knowledge this is the first systematic documentation of the longitudinal clinical variables associated with AKI-ACI, with comparison with AKI, ACI, and NI in COVID-19.

## Methods

### Study Design, Population, and Data Source

This retrospective study was approved by the Einstein-Montefiore Institutional Review Board (#2020-11389). All patients in this study were seen in The Montefiore Health System (MHS) and tested for SARS-CoV-2 infection using real-time polymerase chain reaction test (RT-PCR) on a nasopharyngeal swab between January 1, 2020, and January 29, 2021. The Montefiore Health System is one of the largest healthcare systems in New York City with 15 hospitals located in the Bronx, the lower Hudson Valley, and Westchester County serving a large, low-income, and racially and ethnically diverse population that was hit hard by COVID-19 early in the pandemic (13, 26).

Health data were searched and extracted as described previously (13, 27). In short, de-identified data were made available for research by the Montefiore Einstein Center for Health Data Innovations after standardization to the Observational Medical Outcomes Partnership (OMOP) Common Data Model (CDM) version 6. OMOP CDM represents healthcare data from diverse sources, which are stored in standard vocabulary concepts (28), allowing for the systematic analysis of disparate observational databases, including data from the electronic medical record system, administrative claims, and disease classifications systems (e.g., ICD-10, SNOWMED, LOINC, etc.). ATLAS, a web-based tool developed by the Observational Health Data Sciences and Informatics (OHDSI) community that enables navigation of patient-level, observational data in the CDM format, was used to search vocabulary concepts and facilitate cohort building. Data were subsequently exported and queried as SQLite database files using the DB Browser for SQLite (version 3.12.0).

The primary study outcome was in-hospital mortality as extracted from electronic medical record. Demographic data included age, sex, ethnicity, and race. Chronic comorbidities included obesity, diabetes, congestive heart failure (CHF), chronic kidney disease (CKD), coronary artery disease (CAD), chronic obstructive pulmonary disease (COPD), and asthma. Longitudinal laboratory tests and vitals included creatinine (Cr), estimated glomerular filtration rate (eGFR), albumin, alanine aminotransferase (ALT), aspartate aminotransferase (AST), brain natriuretic peptide (BNP), C-reactive protein (CRP), D-dimer (DDIM), ferritin (FERR), lactate dehydrogenase (LDH), lymphocytes (LYMPH), troponin-T (TNT), white blood cells (WBC), fibrinogen, eosinophils, basophils, neutrophils, prothrombin time (PT), systolic blood pressure (SBP), body temperature, heart rate (HR), and pulse oximetry.

### AKI and ACI Definitions

AKI was defined using the Kidney Disease Improving Global Outcomes criteria as either a 0.3 mg/dl increase in serum creatinine within 48 h or a 1.5x increase in serum creatinine within a 7-day iterative window. The baseline creatinine was determined as the mean of all serum creatinine values 8–365 days preceding hospitalization (20, 21, 29). For patients who did not have creatinine baseline values, the lowest creatinine value during hospitalization was used as the baseline creatinine (19, 30). Urine output was not used to define AKI due to significant missing data. ACI was defined using the 4th Universal Definition of Myocardial Infarction, with a high-sensitivity troponin T level above the 99th-percentile upper reference limit (0.0141 ng/mL) (31–33).

Patients without AKI or ACI were assigned to the no injury group. Note that we also evaluated isolated liver injury and found 713 patients had elevated liver enzymes [AST > 1ULN (>40U/L) and ALT > 1ULN (>35U/L)] (34).

**Figure 1:**
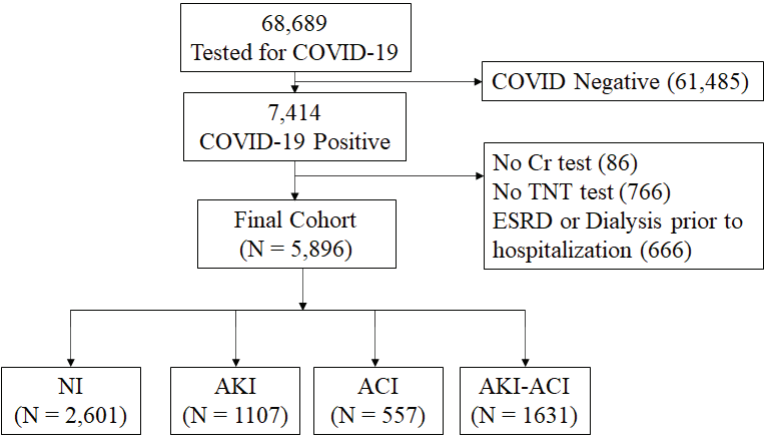
Flowchart of hospitalized patient selection. From March 11, 2020 to January 29, 2021, there were a total of 68,689 hospitalized patients had tests for COVID-19 and 7,414 had a positive COVID-19 test. Cr, creatinine; TNT, troponin-T; NI, no injury; AKI, acute kidney injury; ACI, acute myocardial injury; ESRD, end-stage renal disease.

From March 11, 2020 to January 29, 2021 (Figure 1), there were a total of 68,689 hospitalized patients were tested for COVID-19 and 7,414 had a positive COVID-19 test. Patients who were not hospitalized were excluded. Patients missing Cr or TNT data, and patients with ESKD on dialysis were excluded. This left 5,896 hospitalized COVID-19 patients for the final analysis. Of these, 2,601 had NI, 1,107 had AKI only, 557 had ACI only and 1,631 had AKI-ACI. There were no statistically significant differences in major baseline characteristics (i.e., age, gender, race, ethnicity, and comorbidities) between the included and excluded patients (*p* > 0.05).

### Prediction of AKI, ACI, and AKI-ACI

Logistic regression models were used to rank the importance of clinical variables (demographics, comorbidities, vitals, and blood tests) and predict AKI, ACI, and AKI-ACI onsets using data at admission. Prediction of mortality was also performed using logistic regression. Performance was evaluated using the area under the curve (AUC) of the receiver operating characteristic (ROC) curve with 5-fold cross validation. Note that Cr and TNT, which were used to define AKI and ACI onset respectively, were included in the predictive models because their quantitative values at different days could be predictive of outcomes.

### Temporal Profiles of Clinical Variables

Clinical variables were collected 5 days prior to outcome (death or hospital discharge). Temporal progression of clinical data was time-locked to outcome and compared between groups stratified by survivors and non-survivors. Logistic regression models were used to rank the importance of clinical variables. Prediction performance was evaluated using ROC analysis for individual top variables for different days prior to outcome.

### Statistical Analysis

Statistical analyses were performed using Python and Statistical Analysis System (SAS) software (Cary, NC, USA). Group differences in frequencies and percentages for categorical variables were tested using χ^2^ or Fisher's exact tests. Group comparison of continuous used the non-parametric Kruskal Wallis/ Mann-Whitney *U*-test. Mortality odds ratios (aOR) were adjusted for age, gender, ethnicity, and comorbidities and provided. Differences among AKI-ACI, AKI, ACI, and NI groups for clinical variables in time-series graphs were analyzed *via* linear mixed models and least-squares means. *P* < 0.05 was considered statistically significant and corrected for multiple comparison using the Bonferroni method.

## Results

### Demographics and Comorbidities

The final hospitalized COVID-19 cohort (5,896) consisted of 2,602 (44%) NI patients, 1,107 (19%) AKI-only patients, 557 (9%) ACI-only patients, and 1,631 (28%) combined injury (AKI-ACI) patients. The AKI and ACI incidences were 46.4 and 37.1%, respectively. Table 1 summarizes patient demographics, comorbidities, and laboratory values at admission for each group. The mean ages were 57, 64, 73, and 72 years old in the NI, AKI, ACI, and AKI-ACI groups, respectively (*p* < 0.05 across groups), with ACI or AKI-ACI patients being ~15 years older compared to NI patients (*p* < 0.05). Percentages of female were 54, 48, 41, and 41% in the NI, AKI, ACI, and AKI-ACI groups, respectively (*p* < 0.05 across groups), with ~13% more males in the ACI or AKI-ACI group compared to NI group (*p* < 0.05). There were no group differences across race (*p* > 0.05) and ethnicity (*p* > 0.05).

**Table 1:** Demographics, comorbidities, and laboratory variables at admission of NI, AKI, ACI, and AKI-ACI groups.

	**NI**	**AKI**	**ACI**	**AKI-ACI**	**ACI vs. AKI**	**AKI-ACI vs. AKI**	**AKI-ACI vs. ACI**
***N*** **(%)**	2,601 (44.11%)	1,107 (18.78%)	557 (9.45%)	1,631 (27.66%)			
**Demographics**							
Age in years, mean (SEM)	57.4 (0.4)	63.6 (0.5)	72.7 (0.7)	72.1 (0.4)	^*^	^#^	
Female sex, *n* (%)	1,394 (53.6%)	529 (47.7%)	231 (41.4%)	674 (41.3%)		^#^	
Race, *n* (%)							
White	210 (15.9%)	86 (12.7%)	66 (18.9)	157 (15.0%)			
Black/African American	719 (54.3%)	404 (59.8%)	193 (55.1%)	642 (61.5%)			
Asian	63 (4.8%)	26 (3.8%)	19 (5.4%)	45 (4.3%)			
Other	209 (15.8%)	87 (12.9%)	44 (12.6%)	115 (11.0%)			
Unknown	122 (9.2%)	73 (10.8%)	28 (8.0%)	85 (8.2%)			
Ethnicity, *n* (%)							
Hispanic	1,278 (49.1%)	431 (38.9%)	207 (37.2%)	587 (36.0%)			
Non-Hispanic	1,323 (50.9%)	676 (61.1%)	350 (62.8%)	1,044 (64.0%)			
**Comorbidities**, ***n*** **(%)**							
Hypertension	669 (21.5%)	343 (31.0%)	176 (31.5%)	643 (39.4%)		^#^	^$^
COPD and asthma	259 (10.0%)	91 (8.2%)	52 (9.3%)	136 (8.3%)			
Stroke	44 (1.7%)	28 (2.5%)	16 (2.9%)	79 (4.8%)		^#^	
Diabetes	587 (22.6%)	334 (30.1%)	136 (24.4%)	562 (34.4%)			^$^
Chronic kidney disease	189 (7.3%)	180 (16.3%)	134 (24.1%)	577 (35.4%)	^*^	^#^	^$^
Coronary artery disease	123 (4.7%)	59 (5.3%)	77 (13.8%)	192 (11.8%)	^*^	^#^	
Heart failure	50 (1.9%)	32 (2.9%)	44 (7.9%)	140 (8.6%)	^*^	^#^	
Liver disease	34 (1.3%)	21 (1.9%)	6 (1.0%)	36 (2.7%)			
**Presenting laboratory values, mean, SEM**							
Troponin, ng/mL	0.01 (0.00)	0.01 (0.00)	0.20 (0.04)	0.17 (0.03)	^*^	^#^	
Brain Natriuretic Peptide (pg/mL)	265 (28)	594 (71)	3,343 (288)	3,199 (171)	^*^	^#^	
Creatinine, mg/dL	0.9 (0.01)	1.4 (0.06)	2.3 (0.16)	3.7 (0.23)	^*^	^#^	^$^
eGFR, mg/mL	85 (0.8)	64 (2.3)	43 (2.3)	32 (1.6)	^*^	^#^	^$^
Alanine aminotransferase, U/L	35 (1.0)	35 (2.3)	48 (7.9)	74 (13.9)		^#^	^$^
Aspartate aminotransferase, U/L	39 (1.0)	50 (2.7)	73 (10.8)	102 (16.9)		^#^	^$^
C-reactive protein, mg/dL	6 (0.32)	11 (0.87)	13 (1.11)	16 (0.81)		^#^	
D-dimer, ug/mL	1.4 (0.10)	3.6 (0.44)	5.6 (0.60)	5.7 (0.43)	^*^	^#^	
Ferritin, ng/mL	554 (35)	1,062 (146)	1,446 (236)	2,137 (505)		^#^	
Lactate dehydrogenase, U/L	327 (6)	454 (17)	492 (36)	543 (25)		^#^	
White blood cell count, x10^9^/L	6.9 (0.14)	8.8 (0.33)	9.5 (0.77)	10.4 (0.36)	^*^	^#^	
Lymphocytes, x10^9^/L	1.5 (0.02)	1.2 (0.06)	1.5 (0.20)	1.3 (0.06)			
Basophil x10^9^/L	0.02 (0.000)	0.02 (0.002)	0.03 (0.002)	0.03 (0.001)			
Neutrophils, x10^9^/L	4.7 (0.08)	6.6 (0.28)	6.6 (0.26)	8.2 (0.27)		^#^	^$^
Eosinophil x10^9^/L	0.07 (0.004)	0.04 (0.009)	0.05 (0.006)	0.03 (0.005)		^#^	
Prothrombin time, s	14 (0.11)	15 (0.26)	16 (0.32)	17 (0.23)	^*^	^#^	
Systolic Blood Pressure, mmHg	132 (0.5)	128 (1.5)	131 (2.1)	122 (1.6)			
Pulse Oximetry (%)	97 (0.08)	96 (0.24)	94 (0.66)	94 (0.46)	^*^	^#^	
Temperature, °F	99 (0.03)	99 (0.09)	99 (0.08)	99 (0.08)			
Heart Rate, bpm	90 (0.5)	91 (1.5)	90 (1.6)	97 (1.4)			
**In-hospital mortality**, ***n*** **(%)**	80 (3.1%)	190 (17.2%)	165 (29.6%)	710 (43.5%)	^*^	^#^	^$^

*Group comparison of categorical variables in frequencies and percentages used chi-squared test or Fisher exact tests. Group comparison of continuous variables in means and SEMs (standard error of means) used the Kruskal Wallis/Mann-Whitney U-tests*.

*COPD, Chronic obstructive pulmonary disease*.

*All values are in n (%) unless otherwise specified. Note that all variables shown of all injury groups were significant compared to those of the NI group*.

*,#,$ *Denote significance in pairwise comparisons*.

Patients with ACI-only had more comorbidities including CKD, CAD, and CHF compared to those with AKI-only (*p* < 0.05). Patients with AKI-ACI had a higher prevalence of hypertension, stroke, CKD, CAD, and CHF than those with AKI-only (*p* < 0.05) and had significantly more hypertension, diabetes, CKD than those with ACI-only (*p* < 0.05).

To assess the relative contribution of covariates on prediction of mortality, we performed a relative weight analysis (36) for the logistic regression. The relative weights of these organ injuries, age, CKD, and heart failure were 74.33, 19.24, 1.58, and 1.24, respectively. The relative weights of other comorbidities and demographics were all <1.

### Markers of Organ Injury

At hospital admission, those with combined AKI-ACI had significantly worse levels of cardiac (TNT, BNP), kidney (Cr, eGFR), liver (ALT, AST), inflammatory/immunological (LDH, neutrophils and others) markers (*p* < 0.05) followed by those with ACI or AKI (*p* < 0.05) compared to those with NI. All laboratory values of the three injury groups were significantly different from NI group (*p* < 0.05), except pulse oximetry.

For between group comparisons, those with ACI had significantly higher levels of TNT, BNP, Cr, D-dimer, WBC, prothrombin time, and lower eGFR and pulse oximetry compared to those with AKI. Patients with AKI-ACI had significantly higher levels of TNT, BNP, Cr, eGFR, ALT, AST, CRP, D-dimer, ferritin, LDH, WBC, neutrophils, eosinophil, prothrombin time, and lower pulse oximetry than those with AKI alone and significantly higher levels of Cr, eGFR, ALT, AST, and neutrophil than those with ACI alone.

### In-hospital Mortality

The unadjusted mortality rates of NI, AKI, ACI, and AKI-ACI were 3.1, 17.2, 29.6, and 43.5%, respectively. Odds ratios for in-hospital mortality with adjustment for sex, age and significantly different comorbidities are summarized in Table 2. AKI-ACI patients had 17-fold higher odds of in-hospital mortality [adjusted OR (aOR) = 17.11, 95% CI = 13.63**–**21.66, *p* < 0.001], ACI patients had 7-fold higher odds of in-hospital mortality (aOR = 7.17, 95% CI = 5.35**–**9.64, *p* < 0.001), and AKI patients had 4.7-fold higher odds of in-hospital mortality (aOR = 4.74, 95% CI = 3.66**–**6.13, *p* < 0.001) compared to the NI cohort.

**Table 2:** Adjusted odds ratios and 95% confidence intervals for in-hospital mortality by group.

	**OR**	**95% CI**	***P***
AKI-ACI (ref = NI)	17.1	13.6–21.7	<0.001
ACI (ref = NI)	7.17	5.35–9.64	<0.001
AKI (ref = NI)	4.74	3.66–6.13	<0.001
AKI-ACI (ref = ACI)	1.98	1.61–2.44	<0.001
AKI-ACI (ref = AKI)	3.68	3.05–4.44	<0.001
ACI (ref = AKI)	1.78	1.39–2.29	<0.001

*Covariates used in logistic regression were age, gender, ethnicity and comorbidities that showed statistically significant differences between groups*.

*AKI, acute kidney injury; ACI, acute cardiac injury; ref, reference; NI, no injury*.

Those with combined AKI-ACI had higher risk of death than ACI alone (aOR = 1.98, 95% CI = 1.61**–**2.44, *p* < 0.001) and AKI alone (aOR = 3.68, 95% CI = 3.05**–**4.44, *p* < 0.001). The ACI group had a higher mortality rate than the AKI group (aOR = 1.78, 95% CI = 1.39**–**2.29, *p* < 0.001).

### AKI and ACI Onset

**Figure 2:**
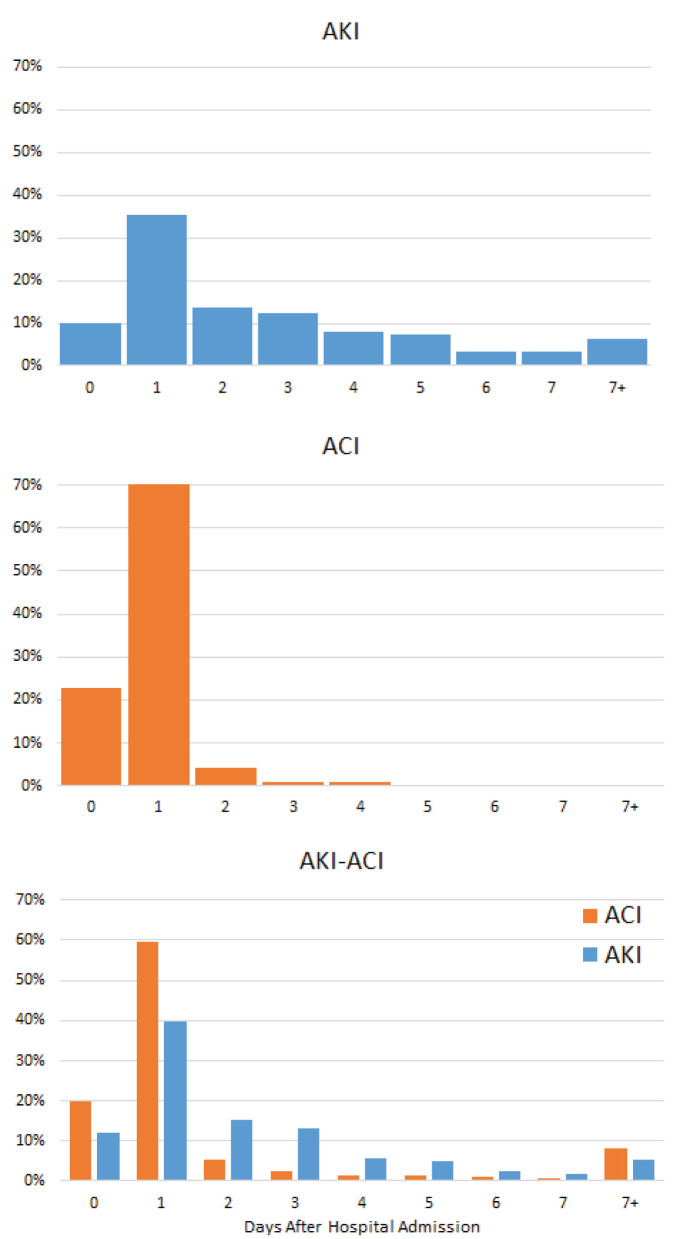
Onsets of AKI and ACI from hospital admission. Percentage of patients who developed AKI, ACI, and AKI-ACI as a function of days after hospital admission.

In the AKI-only group, AKI onset peaked 1 day after hospital admission, but a significant proportion of patients developed AKI throughout the hospitalization (Figure 2). In contrast, in the ACI only group, ACI onset peaked and was predominantly localized to 1 day after admission. In the AKI-ACI group, the onsets of AKI and ACI were similar to those in the AKI-only and ACI-only groups.

### Prediction of AKI, ACI, and AKI-ACI

The top predictors of AKI were Cr, WBC, age, diabetes, and AST, and the predictive model yielded 73 ± 5% accuracy, 93 ± 3% sensitivity, and 27 ± 10% specificity Table 3. The top predictors of ACI were TNT, BNP, Cr, Age, PT, and the predictive model yielded 93 ± 1% accuracy, 96 ± 1% sensitivity, and 82 ± 4% specificity. The top predictors of AKI-ACI were TNT, Cr, DDIM, BNP, PT, and the predictive model yielded 89 ± 2% accuracy, 93 ± 2% sensitivity, and 83 ± 2% specificity.

**Table 3:** Top predictors of AKI, ACI, and AKI+ACI and their performance metrics.

**Cohorts**	**Top predictors**	**Accuracy**	**Sensitivity**	**Specificity**
AKI	Cr, DDIM, LDH, CRP, Neutrophil	0.73 ± 0.05	0.93 ± 0.03	0.27 ± 0.10
ACI	TNT, BNP, Cr, Age, PT	0.93 ± 0.01	0.96 ± 0.01	0.82 ± 0.04
AKI-ACI	TNT, Cr, DDIM, BNP, PT	0.89 ± 0.02	0.93 ± 0.02	0.83 ± 0.02

*Cr, creatine; DDIM, D-dimer; LDH, lactate dehydrogenase; CRP, C-reactive protein; TNT, troponin T; BNP, brain natriuretic peptide; PT, prothrombin time*.

### Temporal Profiles of Clinical Variables

**Figure 3:**
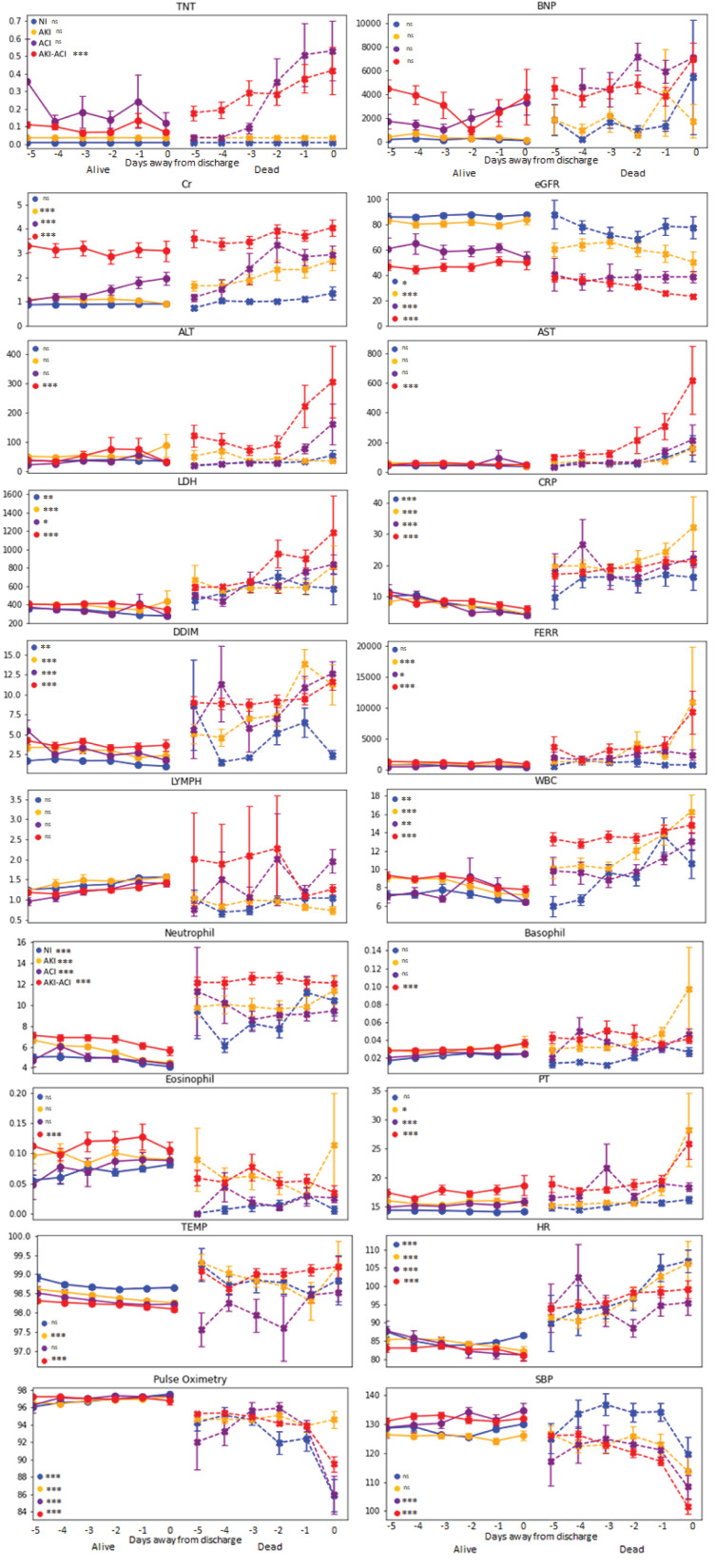
Temporal progression of clinical variables days from outcome. Temporal progression of laboratory tests and vital signs with *t* = 0 representing day of death (for non-survivors) or day of discharge (for survivors). Error bars are SEM. *Indicates *p* < 0.05 between survivors and non-survivors. **Indicates *p* < 0.01 between survivors and non-survivors. ***Indicates *p* < 0.001 between survivors and non-survivors. ns, indicates no significant difference between survivors and non-survivors.

Figure 3 depicts the time series of clinical variables relative to death or discharge for NI, AKI, ACI, and AKI-ACI groups. Overall, laboratory tests at admission were more abnormal, progressively worsened among non-survivors compared to survivors.

For non-survivors, AKI-ACI cardiac (TNT, BNP) and kidney markers (Cr, eGFR) markers were markedly worse days prior compared to the other groups, and liver markers (ALT, AST) markers were markedly elevated and early on only in the AKI-ACI, but not in AKI and ACI group. Furthermore, cell death (LDH), and immunological markers (lymphocyte, WBC, neutrophil, basophil, and eosinophil) were also worse days prior compared to the other groups, whereas inflammatory (CRP, D-dimer, and ferritin) and most vitals were similarly elevated in all groups.

Moreover, the temporal fluctuations of the most of these variables were markedly higher in the AKI-ACI compared to the AKI, ACI, and NI groups. These temporal fluctuations were most noticeable in the non-survivor group.

### Predictors of Mortality

**Figure 4:**
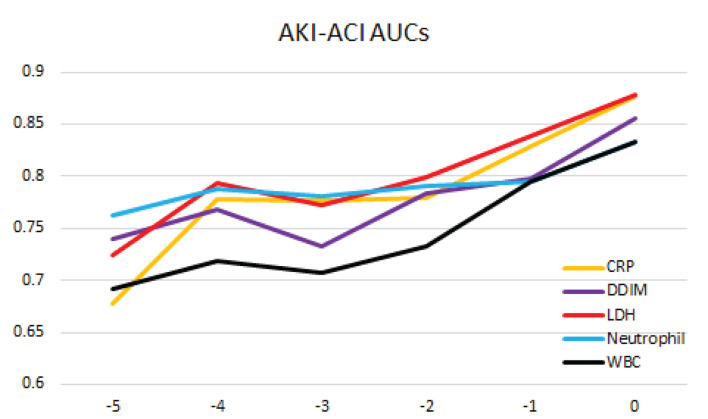
Prediction of mortality likelihood of the AKI-ACI cohort. AUC at different days prior to outcome for individual top predictors and combined top predictors.

The top predictors of mortality in the AKI-ACI cohort were CRP, D-dimer, LDH, neutrophils, and WBC in AKI-ACI cohort. Prediction AUCs were high at days 0 and progressively decreased away from day of outcome, ranging from 0.68 to 0.89 (Figure 4).

## Discussion

This study investigated the clinical characteristics of COVID-19 patients who developed AKI and ACI during hospitalization. ACI onset occurred within a day of hospitalization in contrast to AKI onset which was more distributed across the hospitalization. Patients with AKI-ACI were significantly older, more often men and had significantly more comorbidities compared to those with AKI and NI. COVID-19 patients with AKI-ACI had more elevated levels of cardiac, kidney, liver, inflammatory and immunological markers, followed by those with ACI or AKI compared to those with NI. Patients with AKI-ACI, ACI, and AKI were, respectively, 17.1, 7.2, and 4.7 times more likely to die in the hospital compared to patients with NI. The top clinical predictors of AKI-ACI were TNT, age, Cr, WBC, BNP, and the predictive model yielded 97% accuracy, 94% sensitivity, and 72% specificity. Although physicians already know anecdotally that patients with AKI-ACI have worse outcomes, this study documented the incidence, likelihood of in-hospital mortality using odds ratio and the early clinical laboratory markers that predict which patient will develop AKI-ACI and die in the hospital.

### Incidence of AKI and ACI

We observed a higher incidence of ACI (37.1%) among hospitalized COVID-19 patients compared to previously reported studies with incidences ranging from 16.1 to 23.8% (24). These differences may be explained due to differences in patient populations. Our cohort was minority-predominant and had a relatively high prevalence of cardiovascular comorbidities and lower socioeconomic status that may have been contributing factors to increased adverse cardiovascular outcomes in the setting of COVID-19. We also observed a high incidence of AKI-ACI (28%), suggesting a strong association between AKI and ACI. This is consistent with a previous study that reported an association between AKI and cardiovascular events among COVID-19 patients in the American Heart Association COVID-19 Cardiovascular Disease Registry (37).

### Risk Factors Contributing to AKI and ACI

Compared to those with NI, ACI, and AKI-ACI patients were ~15 years older and had 13% more men. Compared to AKI, ACI, and AKI-ACI patients were ~10 years older and had 6% more men, suggesting that older age and male sex are risk factors for AKI-ACI and ACI. Moreover, preexisting CKD, CAD, CHF, and stroke carried additional risks of developing ACI relative to AKI. The contributions of these additional preexisting cardiovascular comorbidities are not surprising (35, 38). Similarly, preexisting hypertension and diabetes carried additional risk of developing AKI-ACI. Notably, CKD prevalence was remarkably high (35.4%) in the AKI-ACI group compared to only 7.3% in NI, 16.3% in AKI, and 24.1% in ACI group, suggesting that having preexisting CKD markedly increases susceptibility to developing both AKI and ACI.

Having both AKI-ACI signaled a patient is 17.11 times more likely to die in the hospital compared to those without injury, whereas ACI COVID-19 patients were 7.2 times and AKI COVID-19 patients were 4.74 times more likely to die. These observations reflect the multiplicative nature of cardiac and kidney injury on risk of death and that the COVID-19 related cardiovascular event may be the driver of markedly higher mortality.

ACI develops early compared to other organ injuries. The heart may be more susceptible to early damage than other organs as heart muscle has a high density of ACE2 receptors (4, 39). The early ACI onset suggests that ACI is a primary effect of COVID-19, whereas AKI (20, 21) and acute liver injury (40) occur later in the COVID-19 clinical disease course and with more distributed onsets, suggesting that AKI and acute liver injury may arise from secondary effects of COVID-19 (i.e., systemic hypoxia, hypotension, shock, sepsis, and cytokine storm) and/or COVID-19 treatments (6, 7). These secondary effects could also contribute to sustained ACI (41–43). Cardiac injury could lead to AKI or liver injury. Our findings support consideration of pre-emptive and prophylactic treatment early in the disease course and careful monitoring of clinical variables for AKI development.

### Longitudinal Characterization of Clinical Variables Associated With AKI and ACI

Patients with AKI-ACI had markedly worse cardiac, kidney and liver markers days prior to death compared to other groups, suggesting higher incidence of and more severe multi-organ injury. Furthermore, immunological markers were also worse days prior compared to the other groups, whereas inflammatory markers and most vitals were similarly elevated in all groups. These observations indicate AKI-ACI patients had differentially high levels of clinical markers that included more severe multiorgan injuries and overwhelming inflammation and immunological responses.

Significantly elevated TNT and BNP in both ACI and AKI-ACI non-survivor groups 2–3 days prior to death supports a hypothesis of heart attack or heart failure being a possible cause of death in these two groups. In contrast, TNT and BNP were not as elevated in the AKI and NI non-survivor groups. Elevated LDH and CRP seen in non-survivors in all groups are evidence of increased inflammation and immune response to infection. Similarly, elevated WBC in all non-survivors supports sepsis as a possible cause of death in all groups. The steep increase in both ALT and AST in the AKI-ACI non-survivor group points to liver damage close to death and lend evidence to multi-organ failure being a third possible cause of death.

Laboratory variables of the AKI-ACI group were temporally more unstable compared to those with AKI, ACI or NI, especially among non-survivors, suggesting these temporal profiles of clinical variables can also be used to predict mortality (44, 45).

### Predicting of Mortality Associated With AKI and ACI

Understanding the temporal progression of these clinical markers allowed us to construct a prediction model. Longitudinal data accurately predicted mortality likelihood up to 5 days prior. These top predictors of mortality are consistent with a previous report (44) from a different hospital. Most published models used clinical data at admission, not longitudinal variables prior to outcomes (46–49). Prediction using the admission timepoint has relatively poor accuracy compared to a few days prior to outcome. While this finding is intuitively logical, this study provides evidence that our current model can yield a highly accurate prediction a few days prior to the outcome which may lead to earlier recognition, intervention and improvement in clinical outcomes.

### Limitations

A strength of our study is that it addressed multiorgan injury with detailed clinical characteristics in a large diverse population. Our study has several limitations. This is a descriptive retrospective study that could not address the underlying cause of AKI and ACI among hospitalized patients with COVID-19. Missing certain laboratory variables could alter ranking of top predictors. We were unable to analyze how treatment of COVID-19 could have affected AKI and ACI. This study used TNT as indicator of ACI. We were unable to analyze other cardiovascular variables (such as EKGs and echocardiograms) because they would have required manual chart reviews of a large cohort of patients. We also did not study cardiac complications of atrial arrhythmias, ventricular arrhythmias, pericarditis, myocarditis, and heart failure, although this was found to be rare. Although ACI incidence and mortality among COVID-19 patients were generally higher than non-COVID-19 patients, comparison studies controlling for age, race, and ethnicity are needed. This study came from a large population of Black and Hispanic patients and these findings may not be generalizable to other populations. Additional and prospective studies are needed. We did not investigate the effects of anticoagulants on organ injuries (50, 51), the status of the COVID-19 survivors at discharge (52, 53) and the longer-term outcomes (54). As with any retrospective study, there could be unintended patient selection bias and unaccounted confounders.

## Conclusion

A significant number of patients hospitalized with COVID-19 developed combined AKI and ACI. These patients had additional pre-existing risk factors, worse clinical and laboratory variables, markedly worse disease courses, and increased in-hospital mortality. Predictive models using readily available laboratory variables accurately predict which patients are at risk of AKI-ACI and death. Our study has potential clinical implications for hospitalized patients with COVID-19. First, the high incidence of AKI-ACI suggest that AKI-ACI is an important marker of future adverse outcomes in COVID-19. Second, health providers should increase awareness for kidney-cardiovascular complications when AKI-ACI is detected as these complications may assume a lower priority in individuals admitted with COVID-19 given the high respiratory morbidity and mortality of this illness. Third, initiation of kidney and cardiovascular preventive therapies to mitigate kidney and cardiac damages in patients with COVID-19 may be warranted. The ability to identify patients at-risk of developing AKI-ACI early on could enable timely care.

## Data Availability Statement

The original contributions presented in the study are included in the article/supplementary material, further inquiries can be directed to the corresponding author/s.

## Ethics Statement

The studies involving human participants were reviewed and approved by Albert Einstein-Montefiore Institutional Review Board (#2020-11389). Written informed consent for participation was not required for this study in accordance with the national legislation and the institutional requirements.

## Author Contributions

JL: concept, design, collected data, analyzed data, created tables and figures, and drafted paper. AB: concept, design, collected data, analyzed data, and drafted paper. RF and WSH: concept, design, collected data, and edited paper. WH: analyzed data and drafted paper. MF and CR: concept, design, and edited paper. TD: concept, design, supervised, and edited paper. All authors contributed to the article and approved the submitted version.

## Conflict of Interest

The authors declare that the research was conducted in the absence of any commercial or financial relationships that could be construed as a potential conflict of interest.

## Publisher's Note

All claims expressed in this article are solely those of the authors and do not necessarily represent those of their affiliated organizations, or those of the publisher, the editors and the reviewers. Any product that may be evaluated in this article, or claim that may be made by its manufacturer, is not guaranteed or endorsed by the publisher.
